# Strategies and bottlenecks to tackle infodemic in public health: a scoping review

**DOI:** 10.3389/fpubh.2024.1438981

**Published:** 2024-08-14

**Authors:** Andrea Gentili, Leonardo Villani, Tommaso Osti, Valerio Flavio Corona, Angelica Val Gris, Andrea Zaino, Michele Bonacquisti, Lucia De Maio, Vincenzo Solimene, Maria Rosaria Gualano, Carlo Favaretti, Walter Ricciardi, Fidelia Cascini

**Affiliations:** ^1^Section of Hygiene, University Department of Life Sciences and Public Health—Università Cattolica del Sacro Cuore, Rome, Italy; ^2^Saint Camillus International University of Health Sciences, UniCamillus, Rome, Italy; ^3^Leadership in Medicine Research Center, Università Cattolica del Sacro Cuore, Rome, Italy

**Keywords:** infodemic, disinformation, communication, misinformation, recommendations, bottlenecks, public health

## Abstract

**Background:**

The World Health Organization defines “infodemic” as the phenomenon of an uncontrolled spread of information in digital and physical environments during a disease outbreak, causing confusion and risk-taking behaviors that can harm health. The aim of this scoping review is to examine international evidence and identify strategies and bottlenecks to tackle health-related fake news.

**Methods:**

We performed a scoping review of the literature from 1 January 2018 to 26 January 2023 on PubMed, Web of Science, and Scopus electronic databases. We also performed a search of grey literature on institutional websites. The research question has been defined according to the PCC (population, concept, and context) mnemonic for constructing research questions in scoping reviews.

**Results:**

The overall research in the scientific databases yielded a total of 5,516 records. After removing duplicates, and screening the titles, abstracts, and full texts, we included 21 articles from scientific literature. Moreover, 5 documents were retrieved from institutional websites. Based on their content, we decided to group recommendations and bottlenecks into five different and well-defined areas of intervention, which we called strategies: “foster proper communication through the collaboration between science and social media companies and users,” “institutional and regulatory interventions,” “check and debunking,” “increase health literacy,” and “surveillance and monitoring through new digital tools.”

**Conclusion:**

The multidisciplinary creation of standardized toolkits that collect recommendations from the literature and institutions can provide a valid solution to limit the infodemic, increasing the health education of both citizens and health professionals, providing the knowledge to recognize fake news, as well as supporting the creation and validation of AI tools aimed at prebunking and debunking.

## Introduction

Health-related communication is increasingly taking place through digital technologies and social media, especially to provide information on disease etiology, prevention, and treatment, as well as for the promotion of healthy behaviors (1–3). Digital communication is widely available and allows people to access a massive quantity of information on various topics, including public health issues, contributing to the dissemination of both true and false news (4). Despite the great potential of social media to improve general knowledge on many health conditions and their outcomes (1, 2), currently, one of the main challenges with it for health professionals and institutions is related to the dissemination of false or misleading health information (4–6). This phenomenon has historically been present for vaccinations and healthcare emergencies (4, 7, 8) and had adverse effects on people (8). Recently, this situation has intensified, especially during the COVID-19 pandemic (9, 10) when a combination of traditional and new containment strategies was applied in many Countries (11, 12). The spread and accessibility of new technologies, such as social media, have allowed this phenomenon to expand rapidly and exponentially (10). Although there is a potential for positive impacts, the ability to reach many people through digital communication is associated with several potential negative factors, such as misinformation, disinformation, and mistrust (13), due to the spread of information by various sources that could be unverified or uncontrolled, generating an infodemic. The term infodemic was first used by the political scientist Rothkopf (14), during the SARS epidemic to describe the phenomenon of the uncontrolled spread of information, speculation, and rumors amplified by the media. Since then, it was used sporadically by healthcare professionals until the COVID-19 pandemic, when it was highlighted by the World Health Organization (WHO). The WHO defined infodemic as a public emergency, linking the term to the massive dissemination of information (including those considered false and misleading) during an infectious disease outbreak (15). During the COVID-19 pandemic, a tremendous increase in misinformation was observed (9, 16), which greatly impacted the world population’s ability to understand and adapt to the public health measures aimed at containing the pandemic (9, 17) and affecting vaccination campaigns (18). In this context, countering this phenomenon is a priority since it directly affects citizens health and patients’ outcomes, influences the usage of healthcare services, and it may produce an avoidable rise in the burden of certain diseases.

The purpose of this scoping review is to examine international evidence and identify gaps related to the actions taken to counteract the infodemic phenomenon. Specifically, we sought evidence on the recommendations provided and the bottlenecks encountered by the decision-makers and the general population to effectively stop this phenomenon and to limit its consequences on public health.

## Methods

### Defintion of infodemic phenomenon

We performed a scoping review of the literature according to the 5-stage methodological framework described by Arksey and O’Malley (19) for scoping reviews. To define the search string and inclusion criteria, we conducted a preliminary investigation on the concepts and definition of infodemic in public health and its consequences at the population level using institutional sources and literature reviews. For the purpose of this study, we decided to use the definition of infodemic as proposed by Rothkopf (14), which defines infodemic as “a few facts, mixed with fear, speculation, and rumor, amplified and relayed swiftly worldwide by modern information technologies, have affected national and international economies, politics and even security in ways that are utterly disproportionate with the root realities” (14). The decision to use this definition is based on the fact that it appears completer and more comprehensive than others (for example, the WHO includes within its definition only those episodes of misinformation and disinformation concerning infectious diseases).

### Search strategy

**Figure 1:**
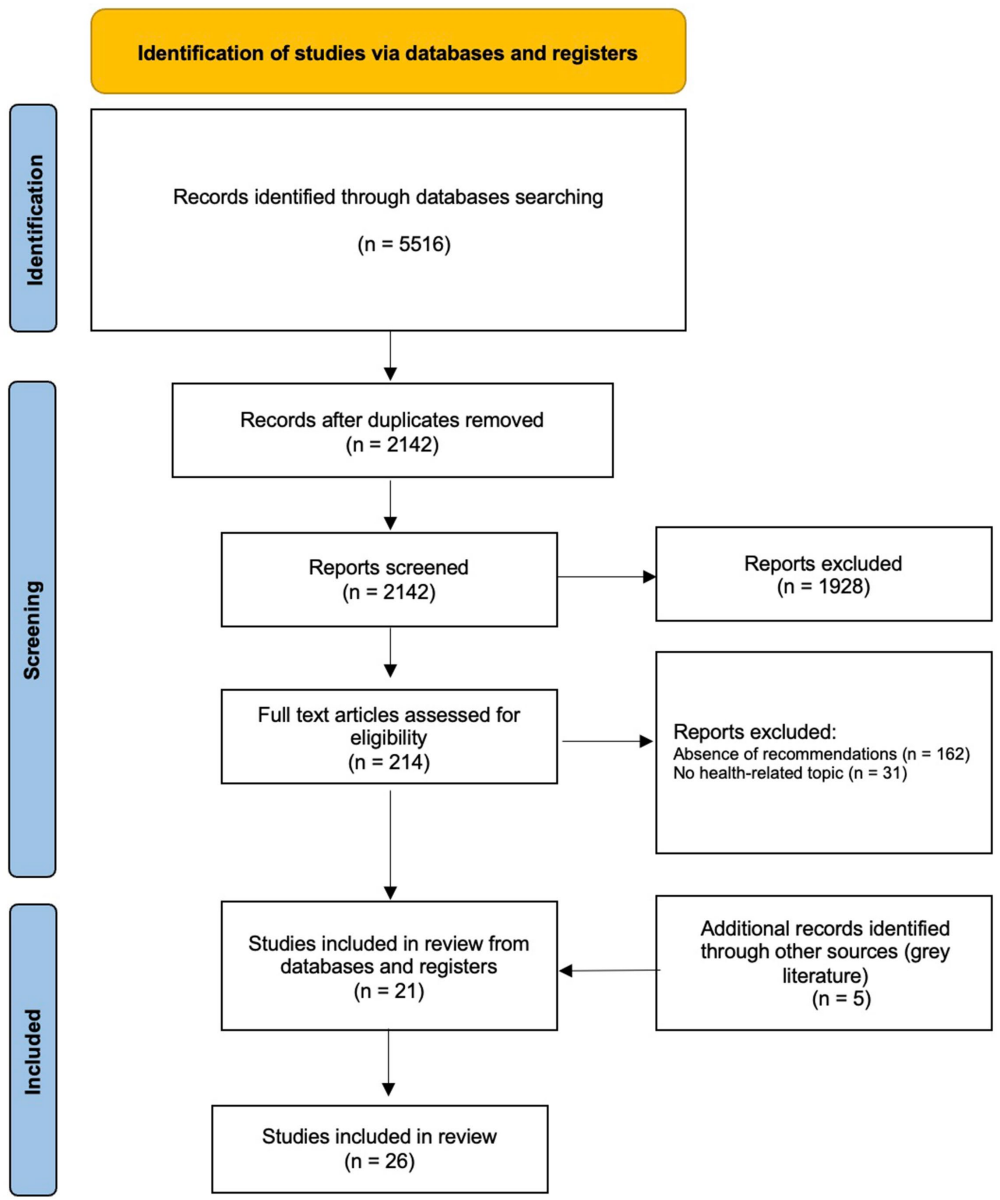
PRISMA for scoping reviews flow diagram (by Moher and colleagues).

The research question has been defined according to the PCC (population, concept, and context) mnemonic for constructing research questions in scoping reviews (20, 21). A systematic search from 1 January 2018 to 26 January 2023 on PubMed, Web of Science, and Scopus electronic databases has been performed using the query reported in Supplementary Datasheet 1. We have also assessed institutional websites (such as the World Health Organization, European Commission, and European Medicines Agency) repeating the query on the Google search engine and analyzing the first 150 results. The review was performed following the Preferred Reporting Items for Systematic Reviews and Meta-Analyses extension for Scoping Reviews (PRISMA-ScR) checklist (22) and results are illustrated in the PRISMA flowchart by Moher and colleagues (Figure 1) (23).

### Eligibility criteria and studies selection

Eligibility criteria of our study have been developed according to the PCC (population, concept, and context) mnemonic for constructing research questions in scoping reviews (Table 1). Our study included peer-reviewed articles written in English that describe structural proposals or recommendations concerning the directions and adoption of interventions or behavior aimed at tackling infodemics on health-related topics. “Health-related topics” was considered any information concerning health risk factors, specific diseases and their characteristics, and any prevention, diagnosis, or treatment topics. We included all types of interventions aimed at supervising, monitoring, preventing, and countering infodemic. In consideration of the research question, opinion pieces, commentaries, and editorials were also considered detectable, so no restrictions have been made based on article type or study design. Instead, research articles and content analyses aimed at estimating the prevalence of mis- or disinformation on health topics without recommendation have been excluded. After the scientific literature search, the identified articles were uploaded on Rayyan software (24), which allowed the removal of all duplicates. Then, each record was screened for title and abstract by at least two researchers independently (LV, AG, AVZ, TO, VFC, AZ, LDM, MB, VS). Then, any disagreement was finally discussed with and resolved by two researchers (LV, AG), when necessary. The pertinent articles with full texts available were then reviewed independently by at least two authors and the articles satisfying the eligibility criteria were included in the scoping review.

**Table 1:** PCC mnemonic framework for research question and eligibility criteria.

P (population)	NA*
C (concept)	Structural proposals or recommendations concerning the directions and adoption of interventions or behavior aimed at tackling infodemics.
C (context)	Any health-related setting.

*Following guidelines there is no need to include this element unless the question focuses on a specific condition or cohort. Our research considers the phenomenon of public health infodemic broadly, not referring to a specific condition/population (21).

### Data extraction, presentation, and categorization

The data from the eligible studies has been extracted in a pre-defined Excel sheet and tested independently on five articles by all the researchers involved in the extraction phase. Nine researchers (LV, AG, AVZ, TO, VFC, AZ, LDM, MB, VS), then extracted data from the papers, ensuring that each article was blindly extracted by two researchers. From each eligible article, we extracted the following information:

1. General information (first author, title, year of publication, country);
2. Health-related topics (vaccinations, COVID-19, etc.);
3. Any specific recommendations aimed at countering the infodemic process in a health-related context;
4. Bottlenecks and gaps experienced: defined, for the porpoise of this work, as any type of structural, technical, social, or political challenge hindering the implementation of an intervention to contrast infodemia;
5. Validation process adopted.

### Data synthesis

The data synthesis process was conducted by four researchers (TO, AVG, LV, AG), employing an iterative process rooted in grounded theory to compare and develop emergent themes (25). Initially, the researchers analyzed the data to identify recommendations aimed at countering infodemic phenomena. These recommendations were then clustered into 24 fundamental statements. This clustering process facilitated the organization of the recommendations into five key strategies, summarized as follows: “foster proper communication through the collaboration between science and social media companies and users.”; “institutional and regulatory interventions”; “check and debunking”; “increase health literacy”; “surveillance and monitoring through new digital tools.”

In addition to recommendations, the bottlenecks identified in each article were also analyzed and clustered. This process resulted in the formulation of 11 fundamental statements, which were then mapped to the corresponding strategies, similar to the recommendations.

The thematic analysis was performed in a double-blind process by the involved researchers.

## Results

The overall research in the scientific databases yielded a total of 5,516 records. After removing duplicates, 2,142 articles were screened based on the title and abstract, and 214 based on full text. Following the inclusion and exclusion criteria, we included 21 articles from scientific literature. Moreover, 5 documents were retrieved from institutional websites. All 26 documents selected during the screening contained a clear and recognizable intervention or directive adopted to limit the spread of health-related infodemic. Considering the health topic, the majority of the included articles were related to COVID-19 (*n* = 15, 57.7%) (26–40), while the others dealt with fake news (41, 42), non-COVID vaccination (43, 44), the control of disinformation in schools (45), risk communication for public health emergencies (46), or with a generic and unclassifiable area of health information (47–51) (Table 2). Data was gathered from a variety of countries, although about 54% of the articles are not placed in any specific geographical setting but produce general considerations (defined as global). The characteristics of the included studies are shown in Table 2.

**Table 2:** Characteristics of the 26 included studies.

First author and year	Type of author	Health topic	Region
Alvarez-Risco, 2020 (36)	Institutional	COVID-19	Peru
Aslani, 2022 (26)	Academic	COVID-19	Global
Au, 2021 (47)	Academic	Health related information	Hong Kong
Benjamin, 2022 (48)	Academic	Generic fake news	Global
Council of Europe, 2022 (45)	Institutional	Infodemic in schools	Europe
Dash, 2021 (32, 41)	Academic	COVID-19	India
Goindani, 2020 (41)	Academic	Generic fake news	Global
Hernandez, 2021 (38)	Academic	COVID-19 vaccines	USA
Jafarzadeh, 2023 (28)	Academic	COVID-19	Global
Office of the US Surgeon General, 2021 (51)	Institutional	Health related information	USA
Ofrin, 2020 (46)	Academic	Risk communication for public health emergencies	South Est Asian Region
Pomeranz, 2021 (27)	Academic	COVID-19	Global
Roozenbeek, 2022 (49)	Academic	Health related information	United Kingdom
Scales, 2023 (29)	Academic	COVID-19 vaccines	Global
Scott J, 2021 (33)	Academic	COVID-19	Global
Song, 2022 (31)	Academic	COVID-19	Global
Steffens, 2019 (43)	Academic	General vaccines	Australia
Tangcharoensathien, 2020 (37)	Institutional	COVID-19	Global
Trethewey, 2020 (50)	Academic	Health related information	UK
UNDP, 2022 (42)	Institutional	Generic fake news	Zambia, Honduras, Kenya, Liberia
Wang, 2022 (35)	Academic	COVID-19	China
Whitehead, 2023 (44)	Academic	General vaccines	Global
WHO, 2020 (40)	Institutional	COVID-19	Global
WHO, 2022 (39)	Institutional	COVID-19	Global
Young, 2021 (34)	Academic	COVID-19	Global
Zhao, 2023 (30)	Academic	COVID-19 vaccines	Global

UNDP, United Nations Development Programme. WHO, World Health Organization.

**Figure 2:**
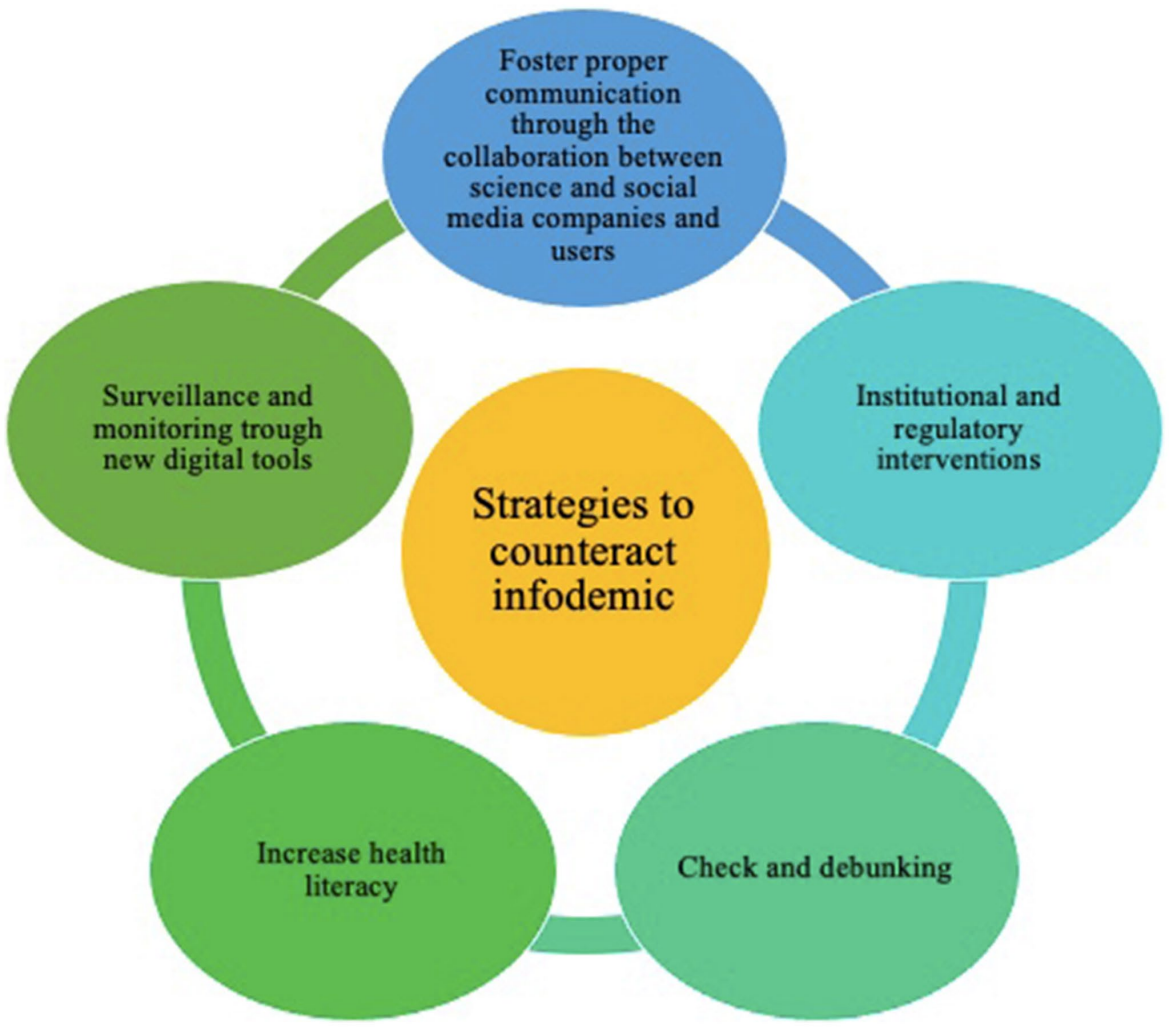
Representation and connections among the different identified strategies to tackle infodemic in public health.

We identified 23 useful recommendations to counter the spread of the infodemic phenomenon. Based on their content, we decided to group those recommendations and bottlenecks into five different and well-defined areas of intervention, which we called “Strategies” (Figure 2). For each of these areas, key actors involved in that specific action, institutions, scientific community, social network services, online services, and platforms were identified and listed. Table 3 summarizes the recommendations, bottlenecks, and actors related to the five different strategies.

**Table 3:** Recommendations, bottlenecks and involved actors per strategy that tackles public health infodemic.

Strategy	Recommendations	Bottlenecks
Foster proper communication through the collaboration between science and social media companies and users	Engagement of social media companies in containing fake news dissemination. Engage amplifiers of society/influencers in disseminating public health information. Promoting safe virtual and physical spaces for people to ask questions. Partnering pro-science community. Incorporate health behaviors in community norms. Avoid many numbers and statistical evidence in messages addressing the public. Social media campaign usage. Define the role and responsibilities of communication partners.	Capacity to address social media and public campaigns Correct identification of people (experts) involved in communication strategies
Institutional and regulatory interventions	Increase public health representatives’ presence on social media/public campaigns. Action from government and authorities. Small financial incentives to foster the dissemination of health-related information. Implementation of laws to tackle the dissemination of fake-news. Establish and infodemic workforce for rapid response	The power of financial incentives may demonstrate a marginal diminishing effect. Legislation that punishes the spread of misinformation does not reduce the intention to share.
Check and debunking	Involvement of experts in fact checking processes. Use of medical and scientific peer-reviewed journals. Apply debunking strategies. Apply prebunking strategies.	Backfire effect of debunking messages. Post-hoc corrections do not reach the same number of people as misinformation. Lack of systematic approach in the debunking strategies. Difficulties in scaling up prebunking strategies.
Increase health literacy	Promoting public health literacy at a population level. Careful dissemination of medical research. Provide examples of correct and incorrect use of science. Tailoring communication strategies in audience specific ways. Train health professionals to better identify/address health misinformation.	Difficulties in structuring health literacy promotion strategies that reach broad segments of the population.
Surveillance and monitoring through new digital tools	Usage of tools to rapidly detect fake news, such as artificial intelligence. Measuring the infodemic and the effectiveness of interventions.	Interaction between different elements of disinformation is not taken into account. Difficulties in the creation and detection of effective indicators for measuring the impact of infodemic at the community level and possible interventions.

### Strategy 1—foster proper communication through the collaboration between science and social media companies and users

Many articles presented one or more recommendations on how to properly communicate and disseminate evidence-based information in the public health sector to avoid the spread of disinformation and misinformation. The most common recommendation was directed at social media companies and the influencers on them (26, 36, 38, 45). Generally, guidance involved requiring stronger actions to contain the dissemination of fake news, and creating a safe, scientifically reliable, and accessible space for people to ask questions and gather public health information through the engagement of experts and influencers in disseminating health-related information (43, 51). In this context, we found that governments and institutions should be strongly involved in countering the dissemination of misinformation through the identification of safe websites, platforms, and communities to respond to the doubts of the citizens as well as defining the role and responsibilities of social media companies and communication partners and require the incorporation of health behaviors in community norms (29, 33, 34, 41, 46). The role of social media, supported by public health experts, relates to the containment of social campaigns that report explicitly erroneous health information, and the engagement of relevant, knowledgeable personalities to communicate proven health information (34, 37, 38, 50). Lastly, the role of the scientific community can be traced back to supporting “pro-science” movements, taking care in building effective science dissemination (43), and avoiding reporting overly technical information to the “lay” public in statistical and scientific terms (31).

### Strategy 2—institutional and regulatory interventions

The recommendations for regulatory interventions are strictly connected to the dissemination of health-related information. We found that governments and other institutional bodies should aim at increasing public health representatives’ presence on social media/public campaigns (26, 36, 50), promoting financial incentives to foster the dissemination of health-related information (47), fostering the adoption of laws to tackle the dissemination of fake news (26, 40), and establishing a specific workforce for rapid response to infodemics (39). We found bottlenecks related to the marginal effect of financial incentives and the possibility that the legislation that punishes the spread of misinformation would not reduce the intention to share this kind of information (47).

### Strategy 3—check and debunking

The ability to rapidly identify fake news requires both technical and public health expertise (26, 32, 48, 49), in accordance with institutional plans to effectively curb the spread of fake news. In this context, the central role of the scientific community emerges. The scientific community needs to be involved in fact-checking activities (26, 48) because it has the important responsibility to work on the obstacles to the implementation of these strategies. For example, by supporting the strategies with systematic methodologies for the dissemination of prebunking (provide citizens with the tools to recognize fake news) and debunking (denying the supposed authenticity of a fake news) strategies. However, we found many bottlenecks related to the implementation of these interventions, such as the backfire effect of debunking messages (30, 49) the lack of an existing systematic approach (33, 34), the capacity to reach people for post-hoc corrections (49), and the difficulties in scaling up prebunking strategies (49).

### Strategy 4—increase health literacy

Most of the articles suggested several recommendations to help the target population to discriminate between true and false or misleading health information. To do this, increasing health literacy for both healthcare professionals and citizens, answering questions in a primary care setting, disseminating evidence-based medical research, providing example of correct and incorrect uses of science, and tailoring communication strategies in audience specific ways were recommended (26–28, 30, 32, 37, 40–46, 48–51). Specific training for healthcare professionals is also proposed (39). For example, some articles (22, 26, 33, 38, 47) recommended improving people’s awareness by promoting long-term public health literacy and encouraging citizens to get vaccinated through evidence-based and trustworthy information. Several studies (24, 28, 39, 40, 45) suggested to strengthen the engagement of communities by supporting them with informational materials and promoting the creation of specific courses to detect health misinformation.

Finally, the importance of protecting expression, disseminating factual information, and ensuring strong protections for whistleblowers was highlighted (23, 42, 44). Some articles (26, 39) identified also bottlenecks related to the distrust of the general population towards political and public health institutions and to the difficulty of influencing personal behaviors.

### Strategy 5—surveillance and monitoring through new digital tools

Many articles recommended the development and use of new digital and artificial intelligence technologies to quickly detect the dissemination of fake news (26, 28, 30, 35, 36, 38, 46, 51), as well as the effectiveness of mitigation measures (37, 40). In particular, the interventions were oriented at:

- Using algorithms and manual methods to effectively and quickly identify misinformation and accurately push the content of rumors through algorithms to minimize their impact is an effective methodology (26, 31);
- Using artificial intelligence approaches, such as a deep convolutional neural network (FNDNet), for the automatic detection of fake news (22, 32, 48); and
- Implementing online tools for fact-checking information (24, 41, 47).

Bottlenecks for this strategy were related to the difficulties of distinguishing and categorizing the certainty of source, as well as the difficulties in the creation and detection of effective indicators for measuring the impact of interventions aimed at reducing infodemic at the community level (26, 32).

## Discussion

In this scoping review, we summarized recommendations and bottlenecks aimed to tackle health-related fake news, misinformation, and disinformation. We identified five main strategies: foster proper communication through the collaboration between science and social media companies and users; institutional and regulatory interventions; check and debunking; increase health literacy; and surveillance and monitoring through new digital tools. The infodemic, defined as the dissemination of an enormous amount of information, often false and misleading (14, 15), currently represents a serious threat to public health, with direct effects on the health both of individuals and the community (8). This phenomenon was particularly evident during the COVID-19 pandemic, in which misinformation and disinformation increased doubts and fear among the population, with reduced adherence to containment measures, vaccination, and treatment while simultaneously promoting the spread of the virus (10, 13, 18). Gathering evidence about possible strategies to mitigate and respond to the infodemic is therefore a public health priority, not only for countering infectious diseases epidemics but for numerous public health issues (52). As a striking example, COVID-19 vaccination reluctance was substantially connected with the frequency, diversity, and usage of social media, as well as with media trust and health information literacy (49). Another example can be found in the news: there was a widespread misconception that drinking high-grade alcohol could both clean the body and eradicate SARS-CoV-2. Following this false information, around 800 individuals have tragically passed, 5,876 have been admitted to hospitals, and 60 have become completely blind after using methanol as a coronavirus remedy (38, 49–52).

The dissemination of rumours, stigmas, and conspiracy theories can have an impact on society as a whole, including the healthcare systems (9). Therefore, it is evident that fake news producers have the power to weaken public confidence in authorities and global health organizations (9), and they have been identified by international health organizations, like the WHO, as new dangers to pandemic preparedness and management. As a result, these organizations have advocated for rigorous surveillance and control measures (53).

The infodemic phenomenon primarily stems from the lack of the general public’s ability, inclination, or time to critically examine most of the content they encounter online, communication management and information dissemination techniques are extremely vital in the field of public health (54). Even when the desire to learn more about a subject related to our own medical condition or symptoms is enhanced, determining the reliability of sources and the accuracy of information is a very challenging task (55). In our review, we identified social media and influencers as vectors that can impact and direct information, both positively and negatively (22, 32). For this reason, it is necessary for institutions to promote collaboration between themselves and social media companies, so that they can convey accurate and reliable information, with the certainty that these derive from established and reliable sources (for example, international organizations). Combining the scientific skills of healthcare professionals with the outreach capabilities of influencers is an indispensable starting point for conveying correct and understandable information to the population. During the COVID-19 pandemic, for example, several countries created a partnership with social influencers to promote vaccination campaigns, with encouraging results (53–57).

Promoting the enactment of laws that contrast the spread of fake news is another way to accomplish this goal (34, 42), even if there is still a very limited international experience on this topic. The production of laws and regulations aimed at countering the dissemination of fake news, as well as the creation of task forces aimed at surveillance and control of this phenomenon could represent valid tools.

The review also emphasized the importance of increasing the health literacy, supporting healthy lifestyles and the dissemination of scientific knowledge. It is known that health literacy relates to a person’s capacity to handle the complex demands of health in contemporary society (56), and it is related to social determinants of health (57). Different studies, for example, have shown that the dissemination of health-related information on social media increased many health outcomes (e.g., quality of life) (58, 59). However, while the dissemination of information through the web is a generally positive factor (allowing, in most cases, the consultation of trusted and surveilled information), the risk of spreading misinformation and disinformation (for example, through Google or other search engines) is high. In this context, a strong government action is needed (60). Given this, it is obvious that health literacy does not serve as a magic bullet to eliminate health inequities, which are primarily caused by the unequal distribution of opportunity, resources, and power (57). However, it is possible to maximize the contribution that health literacy makes to addressing the causes and effects of disparities and enabling individuals to have more control over the factors that affect their health (57).

Certainly, close collaboration between the scientific community, social media companies and influencers (as the main internet actors identified by our review), and governments and institutions can enable the creation of tools aimed at countering infodemic by acting on the five strategies we identified. Similar to other health emergencies (61–63), the multidisciplinary creation of standardized toolkits that collect recommendations from the literature and institutions can provide a valid solution to limit the impact of infodemics, increasing the health education of both citizens and health professionals, providing the knowledge to recognize fake news, as well as supporting the creation and validation of AI tools aimed at prebunking and debunking. It is important to remember that monitoring and correcting the spread of false and misleading news is a priority not only for public health, but for all productive sectors that are negatively influenced by this phenomenon financially.

Our study has several limitations. The adoption of Rothkopf’s (14) definition of “infodemic” may not fully capture modern aspects of the phenomenon, particularly those related to infectious diseases. Moreover, the search strategy was limited to English articles in PubMed, Web of Science, and Scopus, potentially excluding relevant studies in other languages or databases. Additionally, only the first 150 Google results for grey literature were reviewed, possibly missing other important sources. Furthermore, the study did not evaluate the effectiveness of the proposed interventions, which limits the practical implications of the findings. Finally, categorizing data into predefined themes might have constrained the analysis, overlooking nuanced aspects of infodemic.

Of course, the implementation of these toolkits can lead to legal and ethical issues (regarding privacy of patients, the control and management of sensitive information, etc.) (64). Nonetheless, ensuring the health of the population is a priority of governments, and ethical and legal issues can be overcome through the transparent and shared creation of tools aimed at fostering proper communication, increasing health literacy, encouraging recognition and reducing the spread of fake news.
