# Supplementary material for: Strategies and bottlenecks to tackle infodemic in public health: a scoping review
Source: Front Public Health. 2024 Aug 14;12:1438981. doi: 10.3389/fpubh.2024.1438981 (PMC11359844; doi:10.3389/fpubh.2024.1438981)
Supplement: Supplementary file 1 [file Data_Sheet_1.PDF]

Appendix 1. Search string.

("infodemia" OR "infodemic" OR "infodemiology" OR "disinformation" OR "misinformation" OR “fake news”) AND ("tool*" OR "guide*" OR "approach" OR "plan" OR "guidance" OR "handbook" OR "polic*" OR "strateg*" OR "toolkit" OR recommendation*) AND ("health" OR "public health" OR "health workers" OR "healthcare" OR "health professionals") AND "last 5 years"[dp]
